# Wi-Fi Backscatter System with Tag Sensors Using Multi-Antennas for Increased Data Rate and Reliability

**DOI:** 10.3390/s20051314

**Published:** 2020-02-28

**Authors:** Taeoh Kim, Hyobeen Park, Yunho Jung, Seongjoo Lee

**Affiliations:** 1 Department of Information and Communication Engineering, Sejong University, Neungdong-ro, Gwangjin-gu, Seoul 05006, Korea; taeoh@itsoc.sejong.ac.kr (T.K.); hb8971@itsoc.sejong.ac.kr (H.P.); 2 School of Electronics and Information Engineering, Korea Aerospace University, Goyang, Gyeonggi-do 10540, Korea; yjung@kau.ac.kr

**Keywords:** Wi-Fi, Wi-Fi backscatter, multiple antennas, Internet of Things, energy harvesting

## Abstract

In this paper, we propose tag sensor using multi-antennas in a Wi-Fi backscatter system, which results in an improved data rate or reliability of the signal transmitted from a tag sensor to a reader. The existing power level modulation method, which is proposed to improve data rate in a Wi-Fi backscatter system, has low reliability due to the reduced distance between symbols. To address this problem, we propose a Wi-Fi backscatter system that obtains channel diversity by applying multiple antennas. Two backscatter methods are described for improving the data rate or reliability in the proposed system. In addition, we propose three low complexity demodulation methods to address the high computational complexity problem caused by multiple antennas: (1) SET (subcarrier energy-based threshold) method, (2) TCST (tag’s channel state-based threshold) method, and (3) SED (similar Euclidean distance) method. In order to verify the performance of the proposed backscatter method and low complexity demodulation schemes, the 802.11 TGn (task group n) channel model was utilized in simulation. In this paper, the proposed tag sensor structure was compared with existing methods using only sub-channels with a large difference in received CSI (channel state information) values or adopting power-level modulation. The proposed scheme showed about 10 dB better bit error rate (BER) performance and throughput. Also, proposed low complexity demodulation schemes were similar in BER performance with a difference of up to 1 dB and the computational complexity was reduced by up to 60% compared to the existing Euclidean distance method.

## 1. Introduction

Research and development on IoT (Internet of Things) has been actively conducted recently [1,2,3]. Accordingly, powerless or ultra-low-power wireless communications have also been widely proposed because power saving is a very important issue in IoT devices [4,5]. There are various technologies to transmit data through ambient radio frequency (RF) radio signals [6,7,8]. One of these technologies is a Wi-Fi backscatter system released several years ago [9]. The Wi-Fi backscatter communication is a technology that transmits information of a battery-free tag sensor to a Wi-Fi terminal (or reader) by reflecting an RF wave from a Wi-Fi access point (AP). Depending on the tag’s information, the RF wave is reflected differently. The Wi-Fi backscatter tag sensor can reflect or absorb the Wi-Fi RF signal from a Wi-Fi AP with the operating power of several μW and can transmit its information to the Wi-Fi terminal without destroying normal communications between the Wi-Fi terminal and the Wi-Fi AP.

The Wi-Fi backscatter system does not require a dedicated reader unlike a radio frequency identification (RFID) system, and can communicate over longer distance than near field communication (NFC). Moreover, the Wi-Fi backscatter system has a significant advantage of utilizing the existing Wi-Fi infrastructure. The existing Wi-Fi backscatter technology, however, has a low speed of 1 kbps for uplink communication and a small coverage of two meters. In order to solve these issues, various studies were carried out. A Wi-Fi passive backscatter technology can decode backscattered signals using commercial Wi-Fi radios, but requires a dedicated continuous wave signal generator [10]. A BackFi technology can transmit data at the speed of 5 Mbps and 1 Mbps in the region of one meter and five meters, respectively [11]. Since the RFID-based full-duplex backscatter technology was used, however, RF modules of Wi-Fi devices were supposed to be modified, which made it difficult to apply the technology to the existing Wi-Fi infrastructure. A power-level modulation technique used multiple attenuators in a tag sensor to control the reflection strength of Wi-Fi packets and can increase the data rate in the existing Wi-Fi infrastructure [12]. However, the bit error rate (BER) performance deteriorated since the Euclidean distance between symbols decreased as the number of modulation power levels increased.

In this paper, multiple antennas were deployed to the tag sensor in order to improve the throughput or BER performance of uplink communication in Wi-Fi backscatter systems. Unlike the existing algorithm, which only utilize the impedance of a single antenna to increase the data rate, the proposed backscatter method can achieve better performance by controlling the impedance of multiple antennas independently. In general, symbols utilized in Wi-Fi backscatter communications are modulated onto a carrier by the influence of a tag sensor on the channel. In Wi-Fi backscatter communications, the variations on a channel by a tag sensor deeply depends on the demodulation performance. Therefore, the proposed algorithm employing multiple antennas can increase the performance compared to existing methods by easily affecting channel characteristics due to the antenna diversity.

However, as the number of antennas used in Wi-Fi backscatter system increases, computational complexity required to demodulate increases. In this paper, therefore, we propose three additional low complexity demodulation methods to reduce computational complexity. The existing Euclidean distance (ED) method demodulates the signal by comparing the degree of similarity between test patterns obtained from training sequences and channel state information (CSI) received by multiple antennas [12]. This scheme uses all sub-carriers in the symbol and, thus, has enhanced BER performance, but the computational complexity is high. The SET (subcarrier energy-based threshold) method, which is one of the proposed demodulation methods, uses only sub-carriers with high reliability for calculation of Euclidean distance. The TCST (tag’s channel state-based threshold) method selects only sub-carriers with good discrimination among CSI of preamble for calculating Euclidean distance. The SED (similar Euclidean distance) method measures the degree of similarity using absolute values of magnitude difference, which include only an addition operation. The contributions of this paper are as follows.

1. We proposed a MIMO (multiple-input multiple-output) Wi-Fi backscatter system using multi-antennas tag sensor. Since proposed system controls the impedance of multi-antennas independently and reflection gain does not decrease, degree of difference in the CSI pattern between Wi-Fi symbols was maintained. As a result, BER performance did not deteriorate while increasing data rate.
2. Instead of increasing data rate as needed, another backscatter method for improving reliability was presented by turning all switches on/off in multi-antennas tag sensor.
3. Data rate and reliability improved by using multiple antennas, but computational complexity also increased. So, we proposed dedicating three demodulation methods that reduce computational complexity while maintaining BER performance.

Section 2 briefly describes an existing Wi-Fi backscatter communication system. Section 3 describes the proposed multiple-input and multiple-output (MIMO) Wi-Fi backscatter communication method. Section 4 describes the demodulation method applied for the proposed MIMO Wi-Fi backscatter system and improved demodulation methods to reduce computational complexity. In Section 5, we compare the performance and computational complexity of the existing system with the proposed tag sensor with multi-antenna in the Wi-Fi backscatter system. The contributions of this paper are as follows.

## 2. Existing Wi-Fi Backscatter Communication Systems

**Figure 1:**
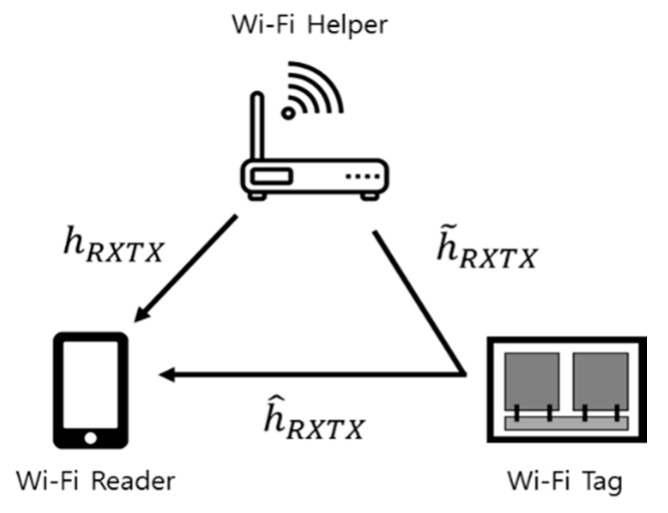
Configuration of the existing Wi-Fi backscatter communication system.

A Wi-Fi backscatter system consists of a Wi-Fi helper (AP), a reader (Wi-Fi device, such as a smart phone), and a tag sensor as shown in Figure 1 [9]. In Wi-Fi backscatter uplink communications, the information is sent from the backscatter tag sensor to the reader. To transmit one bit datum with one (1) value, the tag sensor just reflects the Wi-Fi RF radio wave fully radiated from the Wi-Fi AP during the period of a Wi-Fi packet. The tag sensor can transmit one bit datum with zero (0) value with absorbing the Wi-Fi RF radio packet. Since the tag sensor has to work with very low power or powerlessness, it uses a low-power switch that controls the antenna impedance, which affects the amount of reflection. The analog switch can be implemented in MOSFET (metal-oxide-semiconductor field-effect transistor) technology and can consume less than 1uW [9]. Since the one bit information of tag sensor spans over a Wi-Fi packet period, the impedance of antenna has to be maintained over the period of a single Wi-Fi packet and can be changed for every Wi-Fi packet depending on the information. Consequently, the Wi-Fi backscatter tag sensor embeds its information bits to the Wi-Fi channel without harming normal communications between Wi-Fi devices.

The backscattered signal modulated from the tag sensor changes CSI (in frequency domain) and received a signal strength indicator (RSSI) of a received Wi-Fi packet without directly affecting the data in the Wi-Fi packet. The reader can decode the information of a tag sensor by evaluating the CSI or the RSSI of every received packet. Wi-Fi systems employ an orthogonal frequency division multiplexing (OFDM) technology, and there can be CSI values for 54 sub-carriers in a Wi-Fi packet.

## 3. Proposed Wi-Fi Backscatter System Using Multiple Antennas

In this paper, we propose a method to obtain channel diversity by applying multiple antennas to a Wi-Fi backscatter tag sensor. In the Wi-Fi backscatter system, the tag sensor is a transmitter because it determines the amount of reflection of the Wi-Fi packet by adjusting the antenna’s impedance. Since the number of antennas of the helper (Wi-Fi AP) does not affect the channel diversity, therefore, this paper assumes that the helper works with only one antenna. In the case of a tag sensor, the number of antennas is limited to two because of the size issue caused by the separation distance between the antennas. In this paper, two methods are proposed for increasing the data rate and improving the reliability of the Wi-Fi backscatter system.

The method for increasing data rate uses two antennas of tag sensor (Ant1/Ant2) to send the symbol 0 to the symbol 3 according to the four cases (off/off, on/off, off/on, on/on). In the case of a method for improving the reliability, a symbol of 0 or 1 is transmitted by turning on or off all antennas of tag sensor.

**Figure 2:**
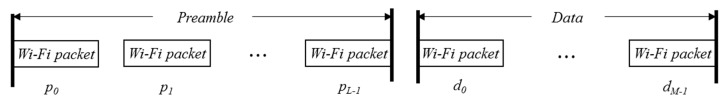
Frame structure in the proposed system.

In Figure 2, we modify the frame structure of the Wi-Fi backscatter system, which uses power level modulation method [12] to suit the proposed system. As shown in Figure 2, the Wi-Fi tag sensor sends L preamble symbols (training sequence) first before data symbol transmission [12] (the tag sensor can tell its data by changing the Wi-Fi channel between a helper and a reader, and the symbol means the tag’s backscatter pattern). Therefore, there is the symbol $x_{k}⊃\left\{\mathrm{preamble}\;p_{l},\;\mathrm{data}\;d_{m}\right\}$ in a frame, and k can be summarized as L+M, where L and M are the numbers of preamble and data backscatter symbols, respectively. The number of preamble backscatter symbols, L, is equal to the number of backscatter symbol types that can be sent. For example, when a tag sensor can send four types of symbols (can backscatter a Wi-Fi packet with four types of gains or patterns from pattern 0 to pattern 3), the number of preamble backscatter symbols is four in the frame.

It is assumed that the path gain $H_{ji}$ between the i-th transmit antenna and the j-th receive antenna is a complex independent identically distributed (iid) Gaussian random variable. It is also assumed that this fading coefficient (path gain) is a quasi-static channel without changing in the symbol interval. The complex baseband representation of the received signal $y_{jk}$ received at the j-th receiver antenna in the k-th symbol interval is given by

(1) $$y_{jk}=\sum_{i=1}^{2}H_{ji}x_{k}+z_{jk}$$

where $x_{k}$ is a normalized complex baseband signal transmitted in the k-th symbol interval and $z_{jk}$ is a Gaussian random noise. The variables in Equation (1) can be expressed as a matrix where $y_{jk}$, $H_{ji}$, $x_{k}$, and $n_{jk}$ are expressed as N×T matrix 𝕪, N×1 matrix H, 1×T matrix 𝕩, and N×T matrix Z, respectively.

(2) 𝕪=H𝕩+Z

N is the number of antennas, and T is symbol interval.

**Figure 3:**
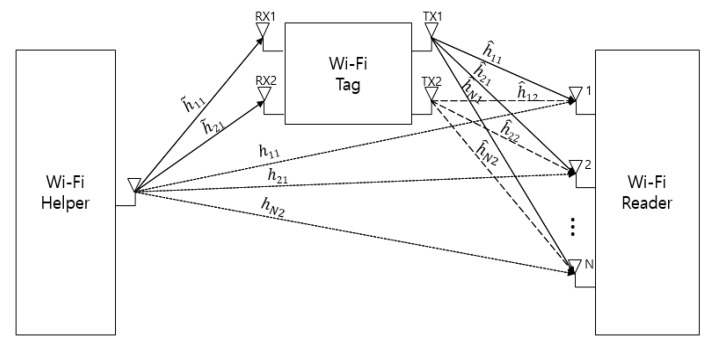
Modeling of channel environments in the proposed system.

For channel H, there are three channel environments, as shown in Figure 1. One is for the path from the helper to the tag sensor, ${\tilde{h}}_{\mathrm{RXTX}}$, another is for the path backscattered by the tag sensor, ${\hat{h}}_{\mathrm{RXTX}}$, and the other is for the path from the helper to the reader, $h_{\mathrm{RXTX}}{}_{HR}$. The channel environments in the proposed system are shown in Figure 3. There is the helper with one antenna, the tag sensor with two antennas, and the reader with N antennas in the proposed MIMO Wi-Fi backscatter system. Figure 3 shows the channel environment of the path from the helper to the reader with the tag sensor backscattering Wi-Fi RF radio waves. When the tag sensor is off, there is only the direct channel environment from the helper to the reader without the tag’s reflection.

Diversity order of the proposed system is expressed according to the number of channel paths. If one antenna is used in reader, the channel environment between tag sensor and reader in the proposed system can be expressed as follows:

(3) $$H=\left[{\tilde{h}}_{11}*{\hat{h}}_{11}+{\tilde{h}}_{21}*{\hat{h}}_{12}\right].$$

In this case, diversity order of proposed system is 2 because there are two channel paths. If N antennas are used in reader, the channel path between tag sensor and reader is expressed as follows:

(4) $$H=\left[\begin{array}{c} \begin{array}{c} {\tilde{h}}_{11}*{\hat{h}}_{11}+{\tilde{h}}_{21}*{\hat{h}}_{12}\\ {\tilde{h}}_{11}*{\hat{h}}_{21}+{\tilde{h}}_{21}*{\hat{h}}_{22}\\ \vdots \end{array}\\ {\tilde{h}}_{11}*{\hat{h}}_{N1}+{\tilde{h}}_{21}*{\hat{h}}_{N2} \end{array}\right]$$

There are 2N channel paths, when the number of antennas used in reader is N. Therefore, diversity order of the proposed system is 2N.

The method of increasing the data rate determines how the antennas of the tag sensor are reflecting depending on the symbol (0 ~ 3) to be sent from the tag sensor. The symbol 0 means turning off reflecting property of all antennas of the tag sensor. For transmitting symbol 1, the first antenna of the tag sensor is ON (fully reflecting an RF wave), and the second antenna is OFF (absorbing an RF wave). For symbol 2, the first antenna is OFF, and the second antenna is ON. Finally, the tag sensor turns on reflecting property of both antennas for the symbol 3. The channel environment, H, with the switch on and off of each antenna is expressed as the following equations.

(5) $$H_{TA_{0}}=\left[\begin{array}{c} h_{11_{HR}}\\ \begin{array}{c} h_{21_{HR}}\\ \vdots \end{array}\\ h_{N1_{HR}} \end{array}\right]$$

(6) $$H_{TA_{1}}=\left[\begin{array}{c} \begin{array}{cc} {\hat{h}}_{11} & 0 \end{array}\\ \begin{array}{c} \begin{array}{cc} {\hat{h}}_{21} & 0 \end{array}\\ \vdots \end{array}\\ \begin{array}{cc} {\hat{h}}_{N1} & 0 \end{array} \end{array}\right]\left[\begin{array}{c} {\tilde{h}}_{11}\\ 0 \end{array}\right]+\left[\begin{array}{c} h_{11_{HR}}\\ \begin{array}{c} h_{21_{HR}}\\ \vdots \end{array}\\ h_{N1_{HR}} \end{array}\right]=\left[\begin{array}{c} {\tilde{h}}_{11}*{\hat{h}}_{11}\\ \begin{array}{c} {\tilde{h}}_{11}*{\hat{h}}_{21}\\ \vdots \end{array}\\ {\tilde{h}}_{11}*{\hat{h}}_{N1} \end{array}\right]+\left[\begin{array}{c} h_{11_{HR}}\\ \begin{array}{c} h_{21_{HR}}\\ \vdots \end{array}\\ h_{N1_{HR}} \end{array}\right]$$

(7) $$H_{TA_{2}}=\left[\begin{array}{c} \begin{array}{cc} 0 & {\hat{h}}_{12} \end{array}\\ \begin{array}{c} \begin{array}{cc} 0 & {\hat{h}}_{22} \end{array}\\ \vdots \end{array}\\ \begin{array}{cc} 0 & {\hat{h}}_{N2} \end{array} \end{array}\right]\left[\begin{array}{c} 0\\ {\tilde{h}}_{21} \end{array}\right]+\left[\begin{array}{c} h_{11_{HR}}\\ \begin{array}{c} h_{21_{HR}}\\ \vdots \end{array}\\ h_{N1_{HR}} \end{array}\right]\;=\;\left[\begin{array}{c} {\tilde{h}}_{21}*{\hat{h}}_{12}\\ \begin{array}{c} {\tilde{h}}_{21}*{\hat{h}}_{22}\\ \vdots \end{array}\\ {\tilde{h}}_{21}*{\hat{h}}_{N2} \end{array}\right]+\left[\begin{array}{c} h_{11_{HR}}\\ \begin{array}{c} h_{21_{HR}}\\ \vdots \end{array}\\ h_{N1_{HR}} \end{array}\right]$$

(8) $$H_{TA_{3}}=\left[\begin{array}{c} \begin{array}{cc} {\hat{h}}_{11} & {\hat{h}}_{12} \end{array}\\ \begin{array}{c} \begin{array}{cc} {\hat{h}}_{21} & {\hat{h}}_{22} \end{array}\\ \vdots \end{array}\\ \begin{array}{cc} {\hat{h}}_{N1} & {\hat{h}}_{N2} \end{array} \end{array}\right]\left[\begin{array}{c} {\tilde{h}}_{11}\\ {\tilde{h}}_{21} \end{array}\right]+\left[\begin{array}{c} h_{11_{HR}}\\ \begin{array}{c} h_{21_{HR}}\\ \vdots \end{array}\\ h_{N1_{HR}} \end{array}\right]=\left[\begin{array}{c} \begin{array}{c} {\tilde{h}}_{11}*{\hat{h}}_{11}+{\tilde{h}}_{21}*{\hat{h}}_{12}\\ {\tilde{h}}_{11}*{\hat{h}}_{21}+{\tilde{h}}_{21}*{\hat{h}}_{22}\\ \vdots \end{array}\\ {\tilde{h}}_{11}*{\hat{h}}_{N1}+{\tilde{h}}_{21}*{\hat{h}}_{N2} \end{array}\right]+\left[\begin{array}{c} h_{11_{HR}}\\ \begin{array}{c} h_{21_{HR}}\\ \vdots \end{array}\\ h_{N1_{HR}} \end{array}\right]$$

where $H_{TA_{0}}$, $H_{TA_{1}}$, $H_{TA_{2}}$, and $H_{TA_{3}}$ are channels when sending symbols 0, 1, 2, and 3, respectively. The reader evaluates the variation of a channel from CSI of the received Wi-Fi packet. The symbol data transmitted from the tag sensor are detected by the reader comparing the received CSI and the prestored CSI data set. Prestored CSI data can be acquired in training sequences prior to tag sensor data communications [11]. This method can transmit two bits per one Wi-Fi packet from symbols 0 to 3, unlike an existing scheme only transmitting one bit for each Wi-Fi packet.

In the second method, the channel diversity effect can be obtained by using multiple antennas of a tag sensor, which can improve the performance, although there is no gain in the data rate. The tag sensor turns all switches of the antennas on and off depending on the data zero (0) and one (1), respectively. In this method, the channel can be defined by

(9) $$H_{TAG_{off}}=\left[\begin{array}{c} h_{11_{HR}}\\ \begin{array}{c} h_{21_{HR}}\\ \vdots \end{array}\\ h_{N1_{HR}} \end{array}\right]$$

(10) $$H_{TAG_{on}}=\left[\begin{array}{c} \begin{array}{cc} {\hat{h}}_{11} & {\hat{h}}_{12} \end{array}\\ \begin{array}{c} \begin{array}{cc} {\hat{h}}_{21} & {\hat{h}}_{22} \end{array}\\ \vdots \end{array}\\ \begin{array}{cc} {\hat{h}}_{N1} & {\hat{h}}_{N2} \end{array} \end{array}\right]\left[\begin{array}{c} {\tilde{h}}_{11}\\ {\tilde{h}}_{21} \end{array}\right]+\left[\begin{array}{c} h_{11_{HR}}\\ \begin{array}{c} h_{21_{HR}}\\ \vdots \end{array}\\ h_{N1_{HR}} \end{array}\right]=\left[\begin{array}{c} \begin{array}{c} {\tilde{h}}_{11}*{\hat{h}}_{11}+{\tilde{h}}_{21}*{\hat{h}}_{12}\\ {\tilde{h}}_{11}*{\hat{h}}_{21}+{\tilde{h}}_{21}*{\hat{h}}_{22}\\ \vdots \end{array}\\ {\tilde{h}}_{11}*{\hat{h}}_{N1}+{\tilde{h}}_{21}*{\hat{h}}_{N2} \end{array}\right]+\left[\begin{array}{c} h_{11_{HR}}\\ \begin{array}{c} h_{21_{HR}}\\ \vdots \end{array}\\ h_{N1_{HR}} \end{array}\right]$$

where $H_{TAG_{off}}$ and $H_{TAG_{on}}$ denote channels when the data zero and one are transmitted, respectively.

In the proposed algorithm, the variation of a channel can be more severe compared to the method employing a single antenna because of antenna diversity. Since the BER performance of a Wi-Fi backscatter system highly relies on the amount of channel variation, the proposed backscatter method can achieve higher BER performance.

## 4. Demodulation Method

We propose a demodulation method for determining a data symbol in a Wi-Fi reader with multiple antennas. To compare the performance along the number of antennas between the system proposed in this paper and the existing system in [12], we first transformed the ED method to suit the proposed system, and we additionally described three low complexity demodulation methods proposed to reduce the computational complexity of ED method.

### 4.1. ED Method for MIMO Wi-Fi Backscatter

The ED method used in the existing Wi-Fi backscatter system is incompatible with the proposed system because the tag sensor uses a single antenna. Therefore, we modified the ED method to fit the proposed system. Demodulation of symbols modulated with channel diversity utilized the Euclidian distance between the CSI data of Wi-Fi packets affected by preamble and data backscatter patterns ($p_{l}$ and $d_{m}$) of the tag sensor. The reader stored L CSI patterns of Wi-Fi packets first received during the preamble duration ($p_{0}$ to $p_{L-1}$) of a Wi-Fi backscatter frame structure for each antenna. When N antennas were employed in the receiver, L × N CSI patterns were saved, and then for the i-th data backscatter symbol ($d_{i}$), the reader calculated L distance between the prestored CSI data and the CSI of the currently received Wi-Fi packet for each antenna. The L distance obtained in each antenna was combined by the preamble backscatter pattern to obtain the total Euclidean distance. That is, when there were L preamble backscatter patterns, L Euclidean distance was calculated for a data backscatter symbol. The Euclidean distance between the m-th data backscatter symbol (CSI pattern) and the l-th preamble backscatter symbol (CSI pattern) is given by

(11) $$q_{l,m}=\;\sum_{j=1}^{N}\sum_{c=0}^{C-1}{\left|p_{j,l}\left(c\right)-d_{j,m}\left(c\right)\right|}^{2}$$

where C is the number of subcarriers assigned to the CSI of Wi-Fi packet, $p_{j,l}$ is the CSI of the Wi-Fi packet received at the j-th antenna in the reader during the period of the l-th preamble backscatter symbol, and $d_{j,m}$ is the CSI of a Wi-Fi packet received at the j-th receive antenna in the reader during the period of the m-th data backscatter symbol. The m-th data backscatter symbol, $r_{m}$, is detected with the shortest Euclidean distance as follows:

(12) $$r_{m}=\underset{l}{\arg}\left(\min\left(q_{l,m}\right)\right)\;(l\;=\;0,\;1,\;\ldots ,\;L-1)$$

where min(∗) is the function that outputs the smallest value. According to the symbol mapping, the bit-level data stream can be restored from the detected symbol, $r_{m}$. For example, when there were L symbol patterns, $\log_{2}L$ data were for each detected symbol. In Wi-Fi backscatter system, the reader used Euclidean distance to compare the similarity between the symbol candidate and the received channel. Determining the degree of similarity was the magnitude of the Euclidean distance, so square root in operation was unnecessary. The square root was omitted for all demodulation methods presented in this paper.

### 4.2. Low Complexity Demodulation Methods

In the reader of existing Wi-Fi backscatter system, the Euclidean distance between the received CSI of preamble and the received CSI of data was utilized for demodulation [12]. Since the Euclidean distance calculation can include meaningless sub-carriers assigned to the CSI, the method of using all sub-carriers may not be effective. The performance of the system was maintained properly, and the computational complexity can be reduced, when operations were performed that contained only good sub-carriers without unnecessary sub-carriers. In order to reduce the computational complexity, we tried various approaches that observed the CSI of received preamble backscatter symbol and used only the appropriate sub-carriers for the operation. We also studied how to use fewer multiplication operations because multiplication operations are more computationally complex than addition operations. As a result, we devised three low complexity demodulation methods.

#### 4.2.1. SET Method

The SET method used only high-energy sub-carriers among the sub-carriers of received data to demodulation. First, we observed the energy (${\left|d_{j,m}\left(c\right)\right|}^{2}$) of the sub-carriers in the CSI of received data and defined the thresholds optimized for each SNR (signal to noise ratio) through simulation. Only subcarriers whose energy exceeded the thresholds was used for Euclidean distance calculation as in the following Equations (13) and (14).

(13) $$e_{j,l,m}\left(c\right)=\{\begin{array}{c} {\left|p_{j,l}\left(c\right)-d_{j,m}\left(c\right)\right|}^{2}\\ 0 \end{array}\begin{array}{c} \mathrm{if}\;{\left|d_{j,m}\left(c\right)\right|}^{2}>threshold\\ Otherwise \end{array}$$

(14) $$q_{l,m}=\overset{N}{\underset{j=1}{\sum }}\overset{C-1}{\underset{c=0}{\sum }}e_{j,l,m}\left(c\right)$$

Thereafter, as in the ED method, we demodulated the data with smallest value of the Euclidean distance between received preamble and data symbol using Equation (12).

#### 4.2.2. TCST Method

If channel state between tag sensor and reader was more independent, Wi-Fi backscatter system can more effectively transmit information of tag sensor to reader. TCST method selected only subcarriers that had highly independent channel state of tag sensor assigned to the CSI and it was used to demodulate symbol data. In a Wi-Fi backscatter system, channel information between the tag sensor and the receiver can be obtained by transmitting preamble before transmitting data [12]. As in the following Equations (15) and (16), the indexes of symbols having maximum received power and the index of symbol having smallest received power were searched for each antenna from the received preambles in j-th antenna.

(15) $$v_{j}=\underset{l}{\arg}\left(\max\left(P_{j,l}\right)\right)$$

(16) $$w_{j}=\underset{l}{\arg}\left(\min\left(P_{j,l}\right)\right)$$

where $P_{j,l}$ is the average received power of CSI of the Wi-Fi packet received at the j-th antenna in the reader during the period of the l-th preamble backscatter symbol. The $v_{j}$ and $w_{j}$ are indexes of preambles which have the largest received power of CSI and the smallest received power of CSI at j-th antenna. We calculated the difference, $z_{j}\left(c\right)$, in magnitude between the two selected preambles, where $z_{j}\left(c\right)$ can be expressed as

(17) $$z_{j}\left(c\right)=\;\left|Re\left\{p_{j,v_{j}}\left(c\right)\right\}-Re\left\{p_{j,w_{j}}\left(c\right)\right\}\right|+\;\left|Im\left\{p_{j,v_{j}}\left(c\right)\right\}-Im\left\{p_{j,w_{j}}\left(c\right)\right\}\right|$$

where $p_{j,v_{j}}$ and $p_{j,w_{j}}$ are the CSI of the Wi-Fi packet received at the j-th antenna in the reader during the period of the $v_{j}$-th preamble backscatter symbol and $w_{j}$-th preamble backscatter symbol. Only subcarriers whose $z_{j}\left(c\right)$ was larger than the threshold were used for Euclidean distance calculation in Equations (18) and (19).

(18) $$e_{j,l,m}\left(c\right)=\{\begin{array}{c} {\left|p_{j,l}\left(c\right)-d_{j,m}\left(c\right)\right|}^{2}\\ 0 \end{array}\begin{array}{c} \mathrm{if}\;z_{j}\left(c\right)>threshold\\ Otherwise \end{array}$$

(19) $$q_{l,m}=\overset{N}{\underset{j=1}{\sum }}\overset{C-1}{\underset{c=0}{\sum }}e_{j,l,m}\left(c\right)$$

Data symbol was demodulated to the smallest value in the Euclidean distance between the preamble symbol and the data symbol by using Equation (12).

#### 4.2.3. SED Method

Since the CSI of received preamble and the CSI of received data were complex numbers, other methods required complex multiplication when calculating the Euclidean distance. Therefore, multiplications in the demodulation process were necessary for each sub-carrier and antenna. Because the multiplication required more instruction clock cycles than the addition, it greatly affected the complexity of the calculation. We proposed a way to compare similarity using only the magnitude difference, not the complex Euclidean distance to solve this problem. A similar Euclidean distance ($q_{l,m})$ can be denoted as

(20) $$q_{l,m}=\;\sum_{j=1}^{N}\sum_{c=0}^{C-1}\;\left|Re\left\{p_{j,l}\left(c\right)\right\}-Re\left\{d_{j,m}\left(c\right)\right\}\right|+\;\left|Im\left\{p_{j,l}\left(c\right)\right\}-Im\left\{d_{j,m}\left(c\right)\right\}\right|.$$

Because additional operations and multiplication were not required to calculate $q_{l,m}$, the SED method had a lower computational complexity than other methods. Data were demodulated to the smallest value among the similar Euclidean distance between the preamble and the data symbol through the Equation (12). But precisely a carrier was demodulated, and symbols were decided/estimated during the demodulation process.

## 5. Experimental Results

In order to verify the performance of the proposed backscatter methods and low complexity demodulation schemes, the system was designed by using MATLAB. The distance between a Wi-Fi helper and a Wi-Fi device (reader) was set to five meters based on the experimental environments of the existing paper [9]. The channel in the simulation followed the model of 802.11 TGn [13]. The 802.11 TGn channel model C and D considered in this paper were both indoor and outdoor environments for wireless LAN (local area network) systems working in the 2 GHz frequency band. Model C is for a large open space (indoor and outdoor), non-line-of-sight (NLOS) conditions, and a 150 ns root-mean-square (rms) delay spread. Model D is for a wide area which is the same environment as the model C, line-of-sight (LOS) conditions, and a 140 ns rms delay spread. The first delay of model D had a Ricean K-factor of 10 dB because of the LOS environment.

There were two different environments for the channel in Wi-Fi backscatter communication systems. The first channel environment was that there were a Wi-Fi helper and a reader without a tag’s reflection. Model C was used for this channel, since a tag sensor did not reflect Wi-Fi RF radio waves and there was a communication channel only between the Wi-Fi helper and the reader. The other environment was that the tag sensor backscatters Wi-Fi RF radio waves while the Wi-Fi helper communicated with the reader. Since LOS existed between the reader and the tag sensor, model D was appropriate for the channel between the reader and the tag sensor with additionally considering the channel effects between the helper and the tag sensor. Therefore, the channel model from the helper to the reader with the backscattering tag sensor used both the delay profiles of models C and D. In addition to that, this paper evaluated the performance of systems with varying the distance between the reader and the tag sensor from one meter to five meters.

### 5.1. Performance Evaluation for MIMO Wi-Fi Backscatter System

In order to compare the BER performance of the proposed MIMO Wi-Fi backscatter system with the power level modulation method [12], we applied the ED method used in the existing system [12] to two systems and used only one antenna for the receiver.

**Figure 4:**
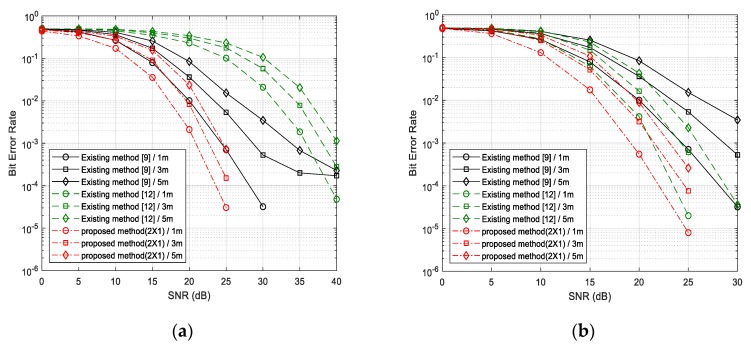
BER (bit error rate) performance for the existing and the proposed backscatter method. (**a**) Proposed MIMO (multiple-input multiple-output) encoding/decoding algorithm for increasing the data rate (the first method); (**b**) proposed MIMO encoding/decoding algorithm for improving the reliability (the second method).

The BER performance of the existing Wi-Fi backscatter systems [9,12] and the proposed MIMO Wi-Fi Backscatter system are shown in Figure 4. Figure 4a shows the performance of the proposed backscatter method adopting the first method to increase the data rate in Equations (5)–(8). An existing method [9] detected the channel variation using a threshold value obtained from the average energy, and performance degradation occurred under high signal to noise ratio (SNR) even with lower data transmission ability. The existing 4-level method [12] had the same data rate as the proposed backscatter method because it transmitted two bits per one symbol by using power-level modulation (backscattering). However, since the reflection gain of 13 and 23 were added to the original gain values, the reflection effects of the tag sensor for Wi-Fi RF radio waves decreased. As a result, the CSI patterns of backscatter symbols were only a little different from one another, which caused performance degradation to occur. Unlike power-level modulation method [12], tag sensor in the proposed system affected effectively the channel environments with multiple antennas’ reflection whose gain was the maximum. Therefore, the presented backscatter methods showed a better BER performance than both algorithms [9,12] regardless of the distance between the reader and the tag sensor. As shown in Figure 4a, the proposed algorithm outperformed the existing 4-level scheme by about 10 dB. Figure 4b shows the BER performance of the proposed backscatter method working as the second mode (refer to Equation (11)) to enhance the reliability (with respect to the BER performance). In this test, the existing method adopting power-level modulation [12] utilized the only 2-level gain (the minimum and the maximum gains) for matching the data speed with the proposed system. Unlike 4-level modulation, the existing method with 2-level modulation showed a good BER performance compared to the other existing method [9] due to the demodulation method with the Euclidean distance. However, this method still cannot defeat the proposed algorithm, since the two antennas of the tag sensor in the proposed system can reflect Wi-Fi RF radio waves simultaneously and the effects on the communication channel between the helper and the reader were much bigger. As shown in Figure 4b, the proposed algorithm had the same BER of $10^{-3}$ under the lower SNR by 5 dB and 3 dB than the existing methods [9] and [12], respectively.

**Figure 5:**
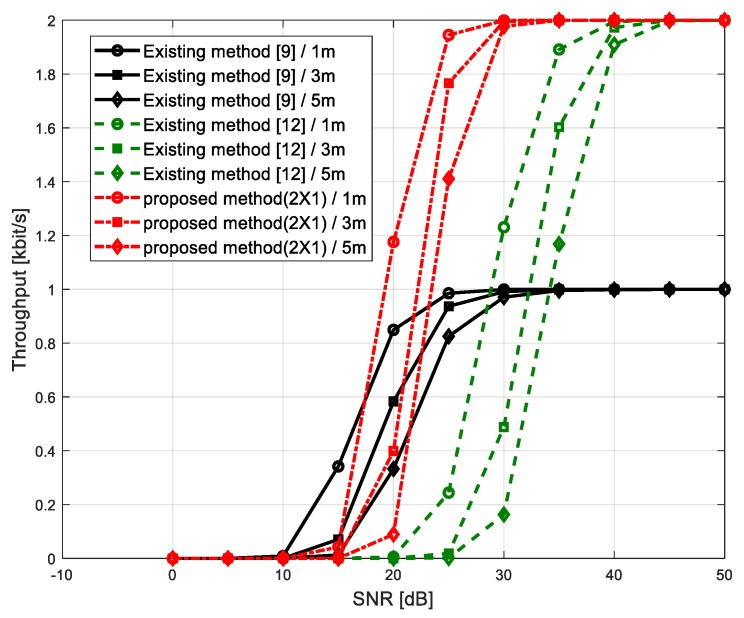
Throughput for existing and the proposed backscatter method.

The throughput of existing and the proposed backscatter systems is shown in Figure 5. The throughput is defined by

(21) $$\mathrm{throughput}=\left(1-P_{FER}\right)\times \alpha \;\;\;\;(\alpha =\log_{2}L\;kbps)$$

where $P_{FER}$ is a frame error rate (FER). In this paper, the FER was obtained when setting a frame length to 128 bits. In Wi-Fi backscatter systems, error correction cannot be performed like radio frequency identification (RFID) because the complicated block cannot be embedded in a tag sensor due to the limited size and the power consumption. Therefore, even the error of a bit in a frame causes the entire error of the frame. As shown in Figure 5, under low SNR conditions (lower than 10 dB), none of the systems can transmit frames without errors because frame errors are even caused by a few error bits. When increasing SNR values, the throughput of the proposed system increased dramatically over other algorithms and shows the maximum performance for SNR conditions over 30 dB regardless of the distance between the reader and the tag sensor. The existing algorithm [9] had a half throughput performance of the proposed system even showing the maximum performance at SNR = 30 dB. The other existing power-level algorithm [12] needed a higher SNR condition by 15 dB for having the same throughput as the proposed backscatter method.

**Figure 6:**
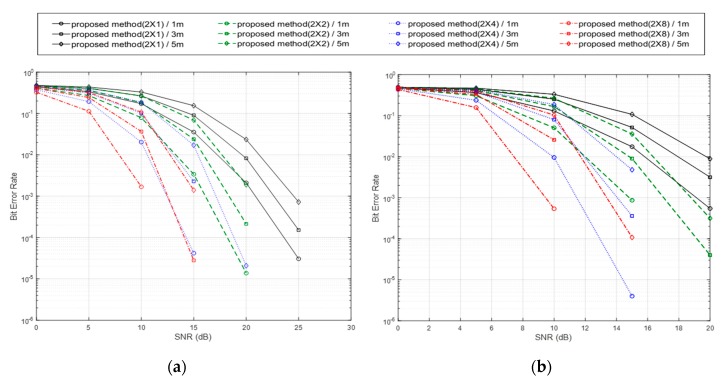
BER performance for the proposed backscatter method depending on the number of antennas of the reader. (**a**) Proposed MIMO encoding/decoding algorithm for increasing the data rate, (**b**) proposed MIMO encoding/decoding algorithm for improving the reliability.

The BER performance in the proposed backscatter method when increasing the number of antennas of the reader from one to eight are shown in Figure 6. Without regarding both the method in the proposed system and the distance between the reader and the tag sensor, the BER performance increased in proportion to the number of antennas employed in the reader. As given in Equation (12), the final Euclidean distance value was calculated from the distance obtained in each antenna and can be obtained from more CSI data with increasing the number of antennas. By increasing the number of antennas in the reader, the reader can gather more information about the channel affected by the tag sensor and can see the difference easily between the tag’s backscatter (reflecting RF waves) and silent (observing RF waves) operations. Since the backscatter system loads the information on the variation of a Wi-Fi communication channel between the helper (AP) and the reader (device), it is very important on the performance to collect the channel information through multiple antennas.

### 5.2. Performance and Computational Complexity of the Low Complexity Demodulation Method

We simulated the case where the antenna of the reader is 1, 2, 4, and 8. Currently, the number of antennas supported in mobile terminals is up to four and research on 8- and 12-antennas array is actively ongoing [14,15]. However, because there were no basic results depending on the number of antennas simulation for eight antennas is performed.

#### 5.2.1. Performance Evaluation of Low Complexity Demodulation Method

**Figure 7:**
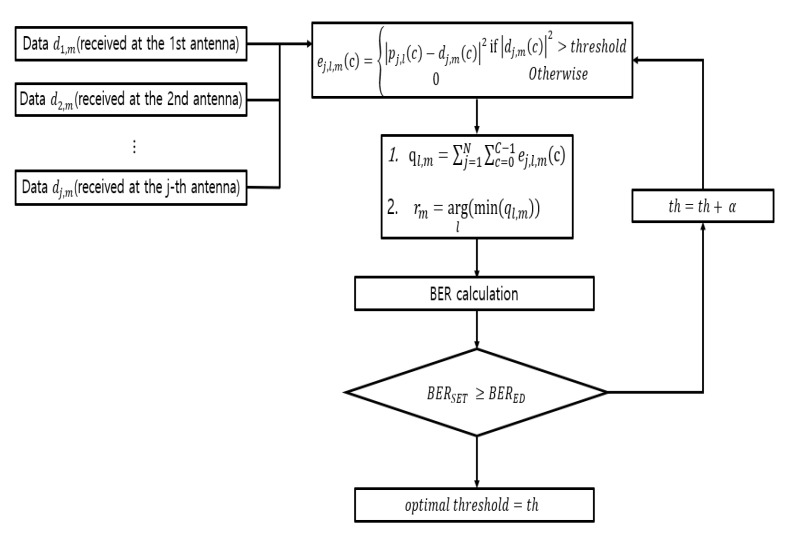
Flowchart for searching the optimal thresholds (SET (subcarrier energy-based threshold) method).

**Figure 8:**
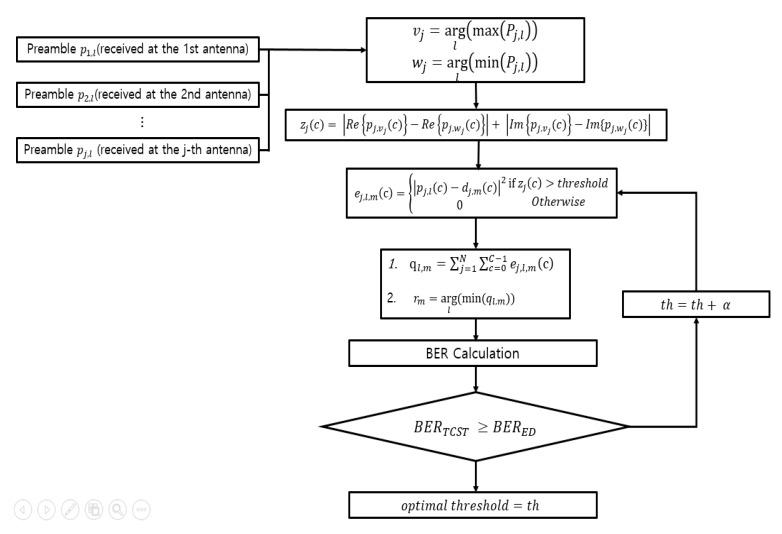
Flowchart for searching the optimal thresholds (TCST (tag’s channel state-based threshold) method).

Since the SET method and the TCST method use thresholds, we first searched optimal thresholds for each SNR and antenna (1, 2, 4, and 8). Here, the optimum threshold meant a maximum value at which performance degradation did not occur when compared with an ED method in a designated SNR environment. Because the number of sub-carriers used for Euclidean distance calculation decreased as the thresholds value increased, it was effective in reducing computational complexity. Figure 7 and Figure 8 show flowcharts for searching optimal thresholds in SET method and TCST method.

In Figure 7, the SET method calculated Euclidean distance using only the sub-carriers whose energy exceeded the thresholds, and m-th data were demodulated to the smallest Euclidean distance ($r_{m})$. Then we calculated the BER and compared it with the BER of ED method.

As shown in Figure 8, the TCST method searched an index of preamble having the maximum received power and an index of preamble data having the smallest received power for each antenna from the received preambles. Subcarriers in which the difference between the selected preamble CSI subcarriers exceeded the threshold were used for demodulation process. After demodulating the data, the BER performance was evaluated and compared with the ED method. SET and TCST methods were repeated until maximum thresholds were found that did not cause performance degradation compared to BER performance of ED method. In the SET method, α= transmitted signal power × 0.1, and in the TCST method, α= 0.1.

Table 1 and Table 2 show the average number of subcarriers applied to the Euclidean distance and optimal threshold for each SNR and antenna in the SET method and TCST method. Regardless of the number of antennas, the higher SNR, the larger threshold and the fewer the average number of subcarriers applied to the Euclidean distance, but the BER performance came out effectively. The thresholds shown in the Table 1 and Table 2 were normalized using the number of antennas and SNR. Also, each threshold was rounded to four decimal places. As the number of antennas increased, the number of sub-carriers used for calculations decreased.

**Figure 9:**
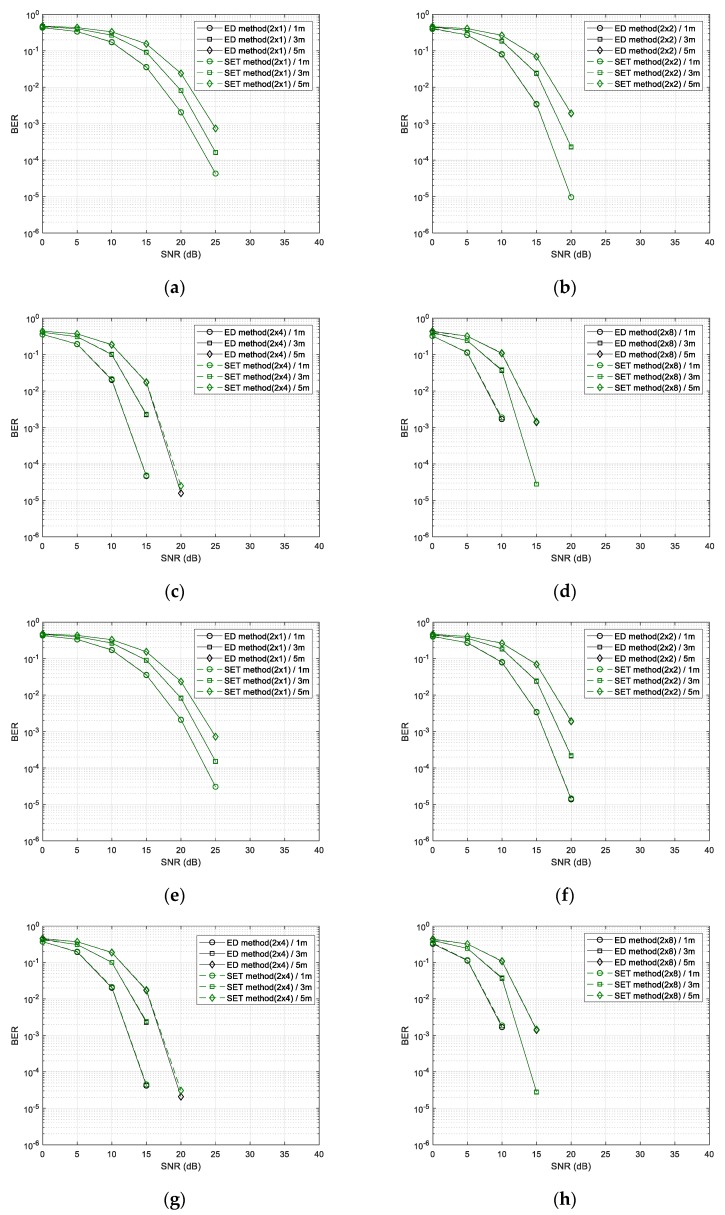
BER performance of SET method for the number of received (Rx) antennas (1, 2, 4, and 8). From (**a**–**d**), when adopting the optimal threshold values with increasing the number of Rx antennas from 1 to 8; from (**e**–**h**) when adopting threshold values calculated from the estimated RSSI (received signal strength indicator) with increasing the number of Rx antennas from 1 to 8.

Figure 9 shows the BER performance of the SET method. Figure 9a–d shows the BER performance of the SET method when applying the optimal thresholds of each SNR and the BER performance of the existing ED method. As shown in Figure 9a–d, when the optimum threshold value was applied, regardless of the number of antennas, we show that the proposed method had almost the same performance as the ED method. In fact, the receiver did not know the ideal SNR, so we used the RSSI to estimate the threshold and compared the method applying estimated threshold with the method applying optimal thresholds. BER performance of two methods is illustrated in Figure 9e–h. There was a difference at about 0.001 dB when comparing the SET method applying the estimated threshold and the SET method applying the optimal threshold.

**Figure 10:**
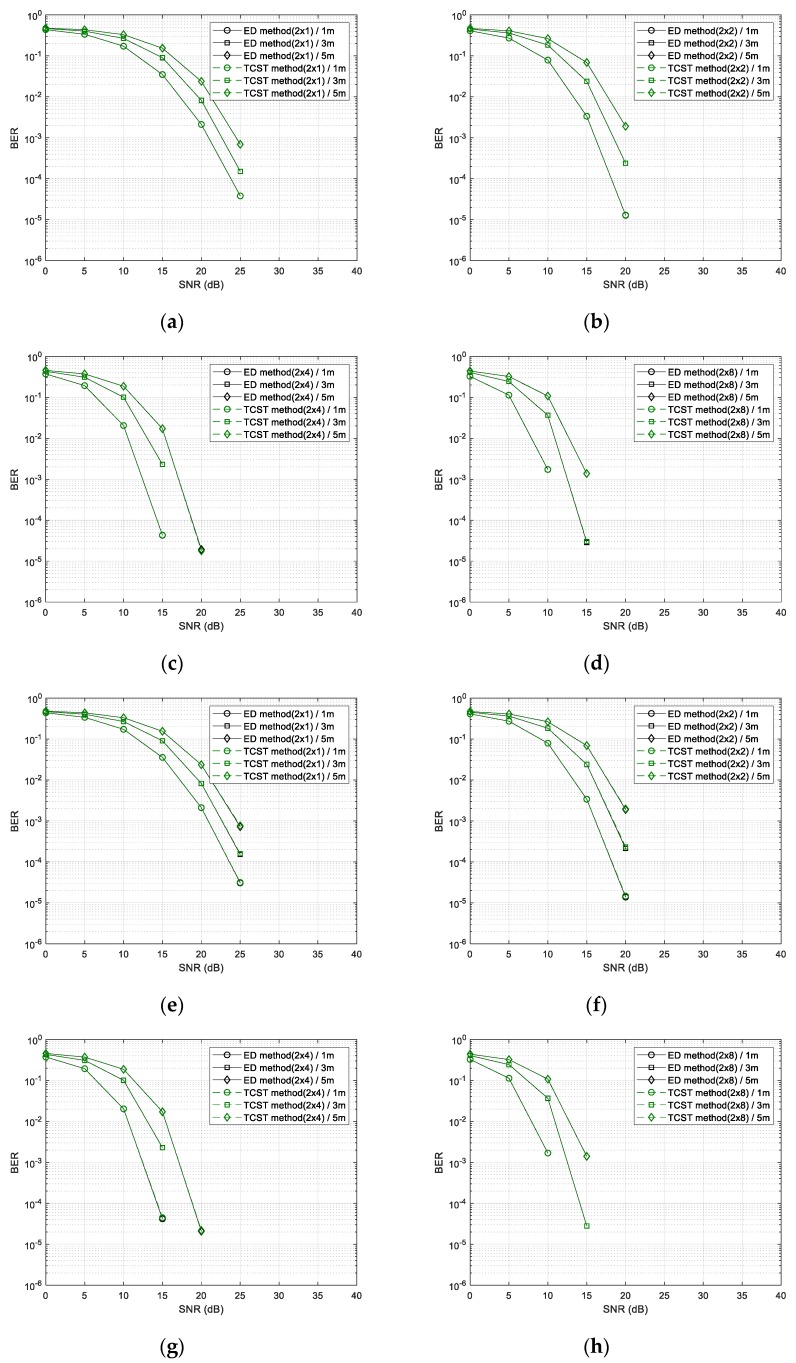
BER performance of TCST method for the number of received (Rx) antennas (1, 2, 4, and 8). From (**a**–**d**) when adopting the optimal threshold values with increasing the number of Rx antennas from 1 to 8; from (**e**–**h**) when adopting threshold values calculated from the estimated RSSI (received signal strength indicator) with increasing the number of Rx antennas from 1 to 8.

Figure 10 shows the BER performance of the TCST method. Figure 10a–d shows the performance of the ED method [12] and the TCST method applying optimal thresholds for each SNR. As shown in the Figure 10a–d, the TCST method when the optimum threshold was applied showed the similar performance as the ED method regardless of the number of antennas. In the Figure 10e–h, we compared the performance of the TCST method applying the optimal threshold and the TCST method applying the threshold estimated via RSSI of the received signal. The TCST method using the estimated threshold has a 0.1 dB performance degradation.

**Figure 11:**
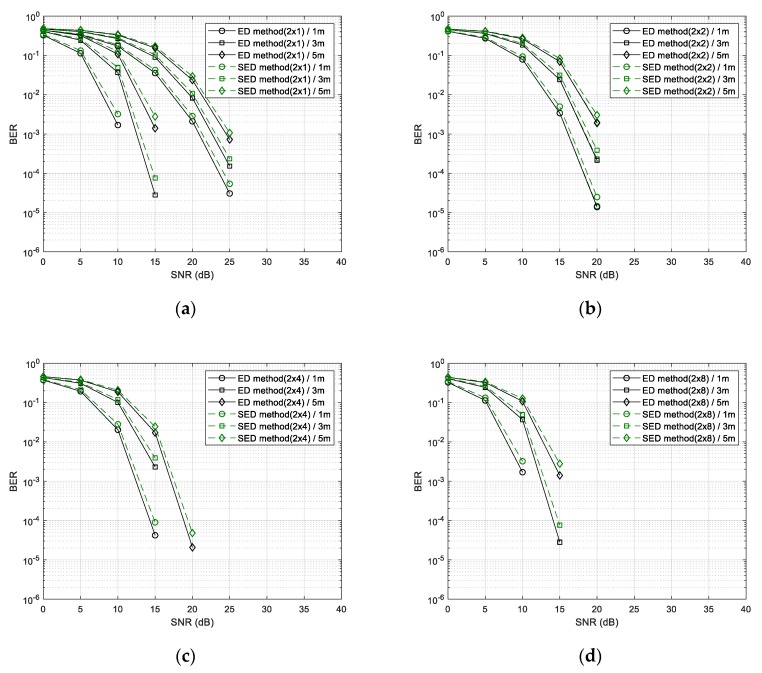
BER performance of SED method for the number of received (Rx) antennas (1, 2, 4, and 8). (**a**) The number of Rx antennas is 1; (**b**) the number of Rx antennas is 2; (**c**) the number of Rx antennas is 4 and (**d**) the number of Rx antennas is 8.

Figure 11 shows the performance when using the ED method and the SED method for data demodulation. The SED method had a performance degradation of 1 dB compared to other methods, but the calculation complexity was the lowest because no multiplication was used in the demodulation process.

#### 5.2.2. Computational Complexity of Proposed Demodulation Methods

In this paper, we compared the computational complexity of the conventional schemes [12] and the proposed demodulation schemes. We calculated the number of instruction clock cycles of each demodulation method using the number of additions and multiplications used in the operation. Table 3 provides formulas for the number of addition and multiplication used in each demodulation method. The formula is expressed in terms of the number of antennas and sub-carriers used in the MIMO Wi-Fi backscatter system. The SED method was not formulated because multiplication operation was not utilized.

Table 4 shows the computational complexity of the demodulation schemes presented in this paper. The number of instruction clock cycles for multiplication was three times larger than the number of instruction clock cycles for the addition. Total number of instruction clock cycle is thus as follows:

(22) $$\begin{array}{c} Total\;\#\;of\;instrucion\;clock\;cycles\\ \;\;\;\;\;\;\;\;\;\;\;\;\;\;\;\;\;\;\;\;\;\;\;\;\;\;=\#\;of\;instruction\;for\;addition\\ \;\;\;\;\;\;\;\;\;\;\;\;\;\;\;\;\;\;\;\;\;\;\;\;\;\;+(3\times \#of\;instruction\;for\;multiplication) \end{array}$$

We calculated computational complexity of the SET method and TCST method applying the estimated threshold. In the case of the SET method and TCST method, the number of sub-carriers applied in the Euclidean distance calculation decreased as the number of antennas used increased. SET method had 7%, 11%, 32%, and 46% reduction in computational complexity compared to the ED method. The complexity of TCST method increased by 3% rather than the ED method when one antenna was used because degree of independence of channel state between tag sensor and reader was calculated. However, as the number of antennas increased, it decreased by 4%, 21%, and 33%. The computation of RSSI used to estimate the threshold was not included in SET and TCST methods because RSSI was included in the Wi-Fi standard. The SED demodulation method had some performance degradation, but there was no multiplication operation that had a higher computational complexity than the addition operation. Therefore, the computational complexity of the SED method was 60% less than that of the ED method and was the largest compared with proposed demodulation methods. We recommend the SET demodulation method, which resembled the performance of the existing methods presented in this paper and had low computational complexity.

## 6. Conclusions

In this paper, two methods to apply multiple antennas to a tag sensor were proposed for increasing the data rate and improving the reliability of the existing Wi-Fi backscatter system. One of the proposed backscatter methods could achieve higher throughput by controlling the impedance of multiple antennas independently. The other switched the gain of multiple antennas in the tag sensor simultaneously to maximize the effects on the communication channel between the helper and the reader and could enhance the BER performance effectively. In order to evaluate the performance of the proposed algorithm with respect to BER and throughput, the system was designed by using MATLAB and was tested under the 802.11 TGn channel models. From the simulation results, the proposed backscatter method showed a better performance in terms of both BER and throughput compared to existing algorithms. The proposed system obtained the maximum gain of 15 dB in SNR with the same throughput as the existing power-level algorithm [12] and the maximum gain of 5 dB in SNR with the same BER as the existing threshold method [9], regardless of the distance between the tag sensor and the reader.

The throughput performance of the proposed system was better than about 10 dB compared to the power level modulation method [12]. In addition, we presented three low complexity demodulation methods to determine received data symbol in MIMO Wi-Fi backscatter system.

The computational complexity, when comparing each method with ED method, was reduced to a maximum of 46%, 32%, 60% and BER performance had a difference of up to 0.001 dB, 0.1 dB, and 1dB. Since BER performance and computational complexity were in a trade-off relationship with each other, an appropriate method can be used according to the system.

The simulation results showed that the proposed backscatter method was very useful for a Wi-Fi backscatter system, in which power saving was an important issue under reliable communication conditions.

## Figures and Tables

**Table 1:** Data used in Euclidean distance calculation and optimal threshold for each SNR (signal to noise ratio) and number of antennas (SET method).

			# of Antennas			
			1	2	4	8
**SNR**	0	Threshold	1	1	1	0.75
		Average # of subcarrier	36	25	13	7
	5	Threshold	0.316	0.158	0.08	0.039
		Average # of subcarrier	45	45	45	45
	10	Threshold	0.1	0.05	0.025	0.125
		Average # of subcarrier	49	50	50	50
	15	Threshold	0.031	0.015	0.007	0.004
		Average # of subcarrier	51	51	51	51
	20	Threshold	0.01	0.005	0.03	0.083
		Average # of subcarrier	51	51	49	38
	25	Threshold	0.003	0.031	0.119	0.193
		Average # of subcarrier	51	50	42	26
	30	Threshold	0.02	0.12	0.25	0.538
		Average # of subcarrier	51	47	33	9
	35	Threshold	0.063	0.198	0.64	0.988
		Average # of subcarrier	50	43	17	3
	40	Threshold	0.08	0.25	1	1
		Average # of subcarrier	49	41	10	3

**Table 2:** Data used in Euclidean distance calculation and optimal threshold for each SNR and number of antennas (TCST method).

			# of Antennas			
			1	2	4	8
**SNR**	0	Threshold	1	0.5	0.25	0.125
		Average # of subcarrier	51	51	51	51
	5	Threshold	0.632	0.158	0.079	0.04
		Average # of subcarrier	51	51	51	51
	10	Threshold	0.2	0.05	0.025	0.013
		Average # of subcarrier	51	51	51	51
	15	Threshold	0.063	0.016	0.008	0.004
		Average # of subcarrier	51	51	51	51
	20	Threshold	0.01	0.005	0.01	0.023
		Average # of subcarrier	51	51	51	37
	25	Threshold	0.003	0.013	0.026	0.019
		Average # of subcarrier	51	50	34	21
	30	Threshold	0.013	0.02	0.018	0.038
		Average # of subcarrier	50	42	28	1
	35	Threshold	0.004	0.014	0.014	0.028
		Average # of subcarrier	48	37	15	1
	40	Threshold	0.008	0.001	0.021	0.013
		Average # of subcarrier	47	31	1	1

**Table 3:** Number of additions and multiplications of demodulation methods.

	# of additions	# of multiplications
ED	4N×(4C−1)	4N×2C
SET	$4N\times \left(4\hat{C}-1\right)$	$4N\times 2\hat{C}$
TCST	$N\times \left(3C+\left(4\times \left(4\hat{C}-1\right)\right)\right)$	$4N\times 2\hat{C}$
SED	4N×(4C−1)	-

where N is the number of antennas, C is the number of all subcarriers assigned to the CSI data, and $\hat{C}$ is the average number of subcarriers applied to the Euclidean distance calculation in the SET method and TCST method. ED: Euclidean distance method; SET: Subcarrier energy based threshold method; TCST: Tag’s channel state based threshold method; SED: Similar Euclidean distance.

**Table 4:** Number of instruction cycles for proposed demodulation methods [16].

# of Antennas	Method	C	$\hat{C}$	Computational Complexity			
				# of Instruction for Additions	# of Instruction for Multiplications	Total # of Instruction Clock Cycles	Reduction Ratio (%)
N=1	ED	52	-	828	416	2076	-
	SET	-	48	764	384	1916	7
	TCST	-	50	952	400	2152	-3
	SED	52	-	828	-	828	60
N=2	ED	52	-	1656	832	4152	-
	SET	-	46	1464	736	3672	11
	TCST	-	46	1776	736	3984	4
	SED	52	-	1656	-	1656	60
N=4	ED	52	-	3312	1664	8304	-
	SET	-	35	2224	1120	5584	32
	TCST	-	37	2976	1184	6528	21
	SED	52	-	3312	-	3312	60
N=8	ED	52	-	6624	3328	16608	-
	SET	-	28	3552	1792	8928	46
	TCST	-	31	5184	1984	11136	33
	SED	52	-	6624	-	6624	60
